# The quality of life of Spanish patients with Huntington's disease measured with H-QoL-I and EQ-5D

**DOI:** 10.3402/jmahp.v4.27356

**Published:** 2016-10-13

**Authors:** Julie Dorey, Emilie Clay, Amine Khemiri, Anis Belhadj, Patricia Trigo Cubillo, Mondher Toumi

**Affiliations:** 1 Creativ-Ceutical, Paris, France; 2 Creativ-Ceutical, Tunis, Tunisia; 3 Neurology Department, Hospital Ramón y Cajal, Madrid, Spain; 4 Faculté de Médecine, Laboratoire de Santé Publique, Aix-Marseille University, Marseille, France

**Keywords:** Huntington's disease, health-related quality of life, instrument, EQ-5D-3L, H-QoL-I, self-reported outcomes, UHDRS independence scale

## Abstract

**Background and objective:**

Huntington's disease (HD) is an inherited neurodegenerative disorder that heavily affects the patient's motor, cognitive, and psychological functions. Yet, very few studies have measured the impact of this disease on the patient's health-related quality of life (HRQoL) with specific and validated instruments. The aim of this study was to explore the impact of HD on the HRQoL of Spanish HD patients using the self-reported, Huntington Quality of Life Instrument (H-QoL-I) and the generic instrument EuroQoL five dimensions (EQ-5D-3L) and thereafter compare the results obtained with the two instruments.

**Methods:**

Fifty-five patients and an equal number of caregivers participated. Patient assessments included the questionnaires of the Huntington Self-Assessment Instrument's four parts: background information assessment, Huntington clinical self-reported instrument, disease-specific HRQoL assessment (H-QoL-I instrument) and Huntington resource utilisation interview, and the EQ-5D-3L questionnaire. Levels of disease severity were also determined based on the Unified Huntington's Disease Rating Scale that was completed by caregivers. Pearson's correlation tests were computed between H-QoL-I and EQ-5D-3L scores.

**Results:**

The scores obtained with the H-QoL-I instrument showed that motor dimension was the most altered followed by the psychological dimension while the social dimension was the least altered. Increase of disease severity resulted in lower patient QoL. The usual activities and anxiety/depression were the most severely altered dimensions according to the EQ-5D-3L results. Mobility was also altered to a great extent while pain was the least altered dimension. All correlations between H-QoL-I and EQ-5D-3L scores were moderate to high and statistically significant (*p*<0.01) with the exception of the correlation between H-QoL-I socialising score and EQ-5D-3L anxiety score. The highest correlations were found between H-QoL-I motor score and three EQ-5D-3L scores: mobility, self-care, and usual activity.

**Conclusions:**

The quality of life of the Spanish HD patients included in this study was severely affected by HD as demonstrated by the results of the generic EQ-5D-3L and the specific H-QoL-I instruments, which showed considerable impact of the disease on the motor and psychological functions. The H-QoL-I instrument was able to discern psychological and motor functioning dimensions that were altered by the disease with more specificity and accuracy than the generic instrument.

The Huntington's disease (HD) is an inherited autosomal dominant neurological disorder that progressively affects the individual's motor, cognitive, and affective functions (1, 2). The progression of HD symptoms is gradual and may be described in two phases: the pre-diagnosis phase and the diagnostic phase (3). In the pre-diagnosis phase, some subtle changes can occur in the patient's personality (irritability, paranoia), cognitive function (memory loss or confusion), or motor function (3). The diagnostic phase involves the onset of major motor disorders, the most specific being the chorea, which is present in two-third of disease cases (1) and appears, first, as slight nervous tics, but then evolves to affect the whole body, including the respiratory muscles, leading to dyspnoea or deglutition disorders. Other motor disorders include balance disorders, oculomotor disturbances, dystonia, hypotonia, bradykinesia, and metabolic abnormalities (2). Individuals also develop cognitive disorders, which manifest as increased difficulty to concentrate, organise ideas, or speak fluently. Difficulty in organising the spatial information is one of the most distinctive cognitive disorders of this disease (2). The patient progressively loses the capacity to perform complex tasks such as working or driving and gradually reaches a state of complete loss of autonomy. All these symptoms are accompanied by major neuropsychiatric disorders. Patients undergo changes in behaviour and mood and may be subject to depression, hallucinations, and suicide in extreme cases (4). These mental and behavioural changes make the situation even more complex for the patient and also for the family that must not only deal with the psychological burden of such a disease and the physical deterioration of the patient but also with the aggressive and paranoiac behaviour of the patient.

The disease progression impacts several dimensions of the patient's health-related quality of life (HRQoL), encompassing psychological, physical, cognitive, and social health (5–7). Very few studies have assessed the patient's HRQoL in HD and fewer studies using HRQoL-validated instruments. In these studies, the HRQoL was assessed by generic instruments, such as the EQ-5D, the SF-36, and the Sickness Impact Profile (SIP) (8, 9), or HD-specific instruments, such as the Huntington's Disease health-related Quality of Life questionnaire (HDQoL) (6) and the Huntington Quality of Life Instrument (H-QoL-I) (10). Most of the studies concluded that the patient's HRQoL was more severely affected by depressive mood (9) and psychosocial (8) or emotional impairment (5) than by motor or cognitive impairment.

Only symptomatic treatments are available for HD, and they are meant to improve the quality of life of patients. It is therefore important to further describe the factors that impact the HRQoL the most and to describe the HRQoL of patients in Spain, which has not been done previously.

The main objective of this study was to evaluate the HRQoL of Spanish HD patients using the self-reported, disease-specific H-QoL-I. A second objective was to compare the results obtained with those obtained from a generic instrument, the EuroQoL five dimensions (EQ-5D-3L).

## Methods

### Study

The current study was part of the European HD burden survey (Euro-HDB), a comprehensive, observational, cross-sectional study on the burden of disease and HRQoL of HD patients (11). The Euro-HDB involved six countries (France, Germany, Italy, Spain, Sweden, and the United Kingdom) and was extended later to Poland and the United States. The objective of the Euro-HDB study was to collect comprehensive information about HD characteristics, namely patients’ clinical characteristics and healthcare resource utilisation, and the HRQoL characteristics of patients and their caregivers.

Spanish participants in the Euro-HDB study were identified and recruited with the help of a neurologist in the hospital ‘Hospital Ramon y Cajal’ in Spain. A non-random convenience sampling was used: patients aged at least 18 years with a well-established diagnosis of HD were asked to participate in the survey and to ask their main caregiver to participate as well. The survey was designed as a cross-sectional, self-reported interview. There was no interference with the patient's usual treatment and more generally no intervention in the patient's life. Patients were asked to complete a questionnaire and send it back. By sending back the questionnaire, they voluntarily consent to participate in the study. An information letter explaining the objectives of the study was sent together with the questionnaire. The patient recruitment for the observational study took place from the first of November 2010 to the end of December 2010. The response rate was roughly 50%.

### Assessments

Patients completed the Huntington Self-Assessment Instrument (HSAI) and the EuroQoL-5D instrument. The caregivers completed the Unified Huntington's Disease Rating Scale (UHDRS) to assess the independence level of patients.

**Fig. 1:**
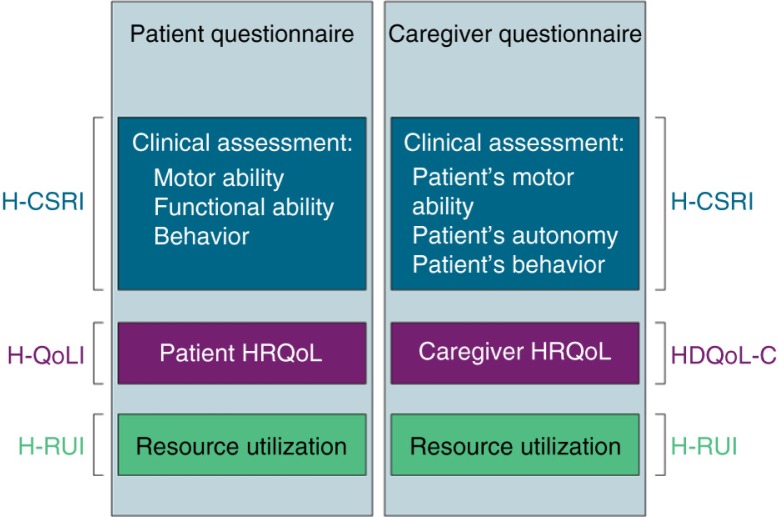
The Huntington Self-Assessment Instrument package.

The HSAI is a comprehensive instrument that assesses all HD characteristics. It consists of two questionnaires: one addressed to the patient and one addressed to his/her caregiver. Both are made up of four parts: background information assessment, the Huntington clinical self-reported instrument (H-CSRI), a disease-specific HRQoL assessment, and the Huntington resource utilisation interview (Fig. 1).

The H-CSRI is the first clinimetric patient-assessed scale for patients with HD. It includes three subscales: motor subscale, including 13 Likert-type items in four dimensions: voluntary movement, stiffness, chorea, precise movement; the functional subscale, including seven Yes/No questions; and the behavioural subscale, including 13 Likert-type items in four dimensions: depression/anxiety, temper, psychotic disorder, and cognition. Higher scores on the function scales indicate more severe symptoms than lower scores. H-CSRI showed satisfactory psychometric proprieties (face validity, IIC, Cronbach's alpha, DIF) and acceptable construct validity. The H-CSRI was already cross-culturally validated for the assessment of the health status of patients with HD in several countries (France, Italy, Germany, Spain, Poland, and the United States) (12).

Patients’ severity was also measured by the independence scale, a scale included in the UHDRS (13). The instrument is a rating scale where a patient's degree of independence is given in percentage (from 0 to 100%) from ‘patient doesn't need special care’ to ‘patient has a tube fed and has a total bed care’ and is rated on 10 score levels, from 0 to 100. Higher scores on the function scales indicate better functioning than lower scores. In this study, caregivers were asked to complete it.

The EuroQoL-5D self-assessment questionnaire measures five dimensions of quality of life: mobility, personal care, routine occupations, pain and discomfort, and anxiety and depression. Each of these domains is noted on three-level Likert-type items: no problem, minor problems, and major problems; so the EQ–5D-3L can capture 243 different health states. Validation of this instrument is very well documented (14–16).

The disease-specific HRQoL assessment of patients was made by the H-QoL-I. It is the first self-reported specific instrument developed to assess the HRQoL of patients with HD. It includes 11 five-point Likert scale items, split into three dimensions: motor functioning (four items), psychology (four items), and socialising (three items). The five response items range from not at all to extremely or very rarely to always. Higher scores on the function scales indicate better HRQoL than lower scores. It demonstrated very good psychometric properties: acceptable construct, external validity, and good reliability (10).

While the EQ-5D instrument was validated and published in Spanish, the instruments specific to HD had been forward-backward translated from French to Spanish and have been then validated (17, 18).

### Data analysis

Impact of HD on patients was described through means and standard deviation (SD) of items and aggregated scores of H-QoL-I and EQ-5D-3L.

### EQ-5D-3L

The 243 combinations of EQ-5D-3L responses were converted to utility scores that might be from −0.594 (a 0 utility score meaning a quality of life as bad as death) to 1 (meaning a perfect full health) by using the UK social tariff (the validated and published algorithm to convert EQ-5D-3L scores to utility scores) (19). Levels of severity were determined based on the UHDRS independence scale: low level of severity included scores 100 and 90 that correspond to stages where patients do not need any help; moderate level included scores 80 and 70 that correspond to stages where patients do not need assistance but stop activities and finally; high level of severity included scores 60, 50, 40, 30, 20, and 10 that correspond to stages where patients need 24-h assistance with their daily living activities. To compare the results of the two HRQoL measures (generic vs. specific), Pearson's correlation was assessed between the dimension scores and the H-QoL-I and the EQ-5D-3L questionnaires.

## Results

A total of 55 patients and as many caregivers participated in the study. Patients’ demographics and disease history are presented in Table 1. Among patients, 27 were males (49%) and 28 were females (51%) with a mean age of 50 years (±14). Forty per cent of patients were workers; the remainder were either unemployed (33%) or retired (27%).

**Table 1:** Patients’ demographics and disease history characteristics

	*N=*55
Demographic	
Male, *n* (%)	27 (49.09%)
Age, years	49.66 (13.66)
Occupational category	
Workers, *n* (%)	18 (40.00%)
Retired, *n* (%)	12 (26.67%)
Unemployed, *n* (%)	15 (33.33%)
Disease history	
Age at onset of symptoms, years	41.25 (16.86)
Age at clinical diagnosis, years	44.12 (13.25)
Tested genetically, *n* (%)	51 (96.23%)

SD, standard deviation. Data are expressed as mean (SD), unless otherwise stated.

On average, patients experienced their first HD symptoms at the age of 41 years (±17) and the disease was diagnosed 3 years later (44 years±13). Almost all patients were tested genetically for HD (96.23%).

The motor, behavioural, and functional H-CSRI scores are given in Table 2. Mean scores for the three dimensions (motor, behavioural, and functional) indicated that precise movement, depression/anxiety, and function were the most altered clinical dimensions.

**Table 2:** Patients’ clinical characteristics

	*N=*55
Motor score	
Voluntary movement score [0–20]	6.33 (5.71)
Stiffness [0–8]	2.20 (2.14)
Chorea [0–16]	5.19 (4.51)
Precise movement [0–8]	4.06 (2.46)
Behavioural score	
Depression anxiety [0–20]	9.12 (5.01)
Temper [0–8]	2.81 (2.18)
Psychotic disorder [0–12]	1.35 (2.16)
Cognition [0–12]	3.52 (2.97)
Functional score	
Functional score [0–7]	3.06 (2.53)

SD, standard deviation. Data from the Huntington Clinical Self-Reported Instrument (H-CSRI) instrument, a clinimetric patient-assessed scale for patients with Huntington's disease (12). Higher scores on the H-CRSI subscales indicate more severe symptoms than lower scores. Data are expressed as mean (SD), unless otherwise stated.

### HD Patients’ HRQoL

#### H-QoL-I scores

**Fig. 2:**
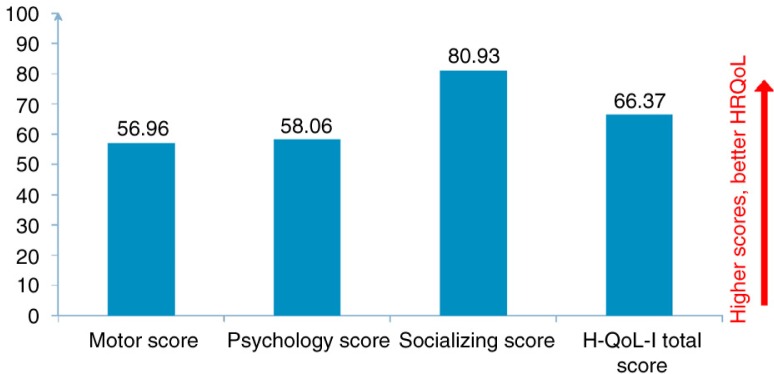
H-QoL-I dimensions and total scores.

Figure 2 shows the scores of the H-QoL-I dimensions as reported by the patients. Motor dimension was the most altered with a mean score of 56.96. Responses by items showed that patients very often or always had difficulties tying their laces (20%), often felt handicapped (29%), or often dropped objects (22%). The psychological dimension was also considerably altered (score: 58.06) with patients feeling often or always powerless (48%) and worrying about symptoms of their disease (38%). Patients had relatively good HRQoL in the social dimension (score 80.93). Most patients reported feeling very rarely or never ignored by people (66.04%), feeling isolated (58%), or no longer being invited (66%) (Fig. 2).

**Fig. 3:**
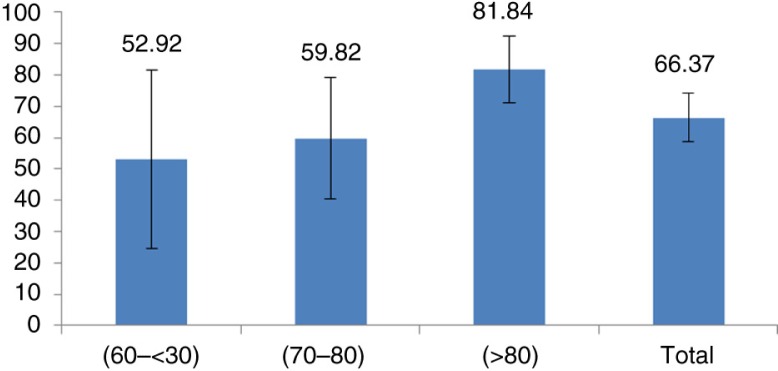
H-QoL-I scores in function of patients’ severity as measured with the independence scale. Severity was categorized as 10–60 for the low independent group, 70–80 for the moderate independent group, and 90–100 for the highly independent group.

H-QoLI scores were found to increase (lower patients’ quality of life) with the increase of disease severity (lower independence scores), ranging from 52.92 for the low severe group to 81.84 for the highly severe group (Fig. 3).

### EQ-5D-3L scores

**Fig. 4:**
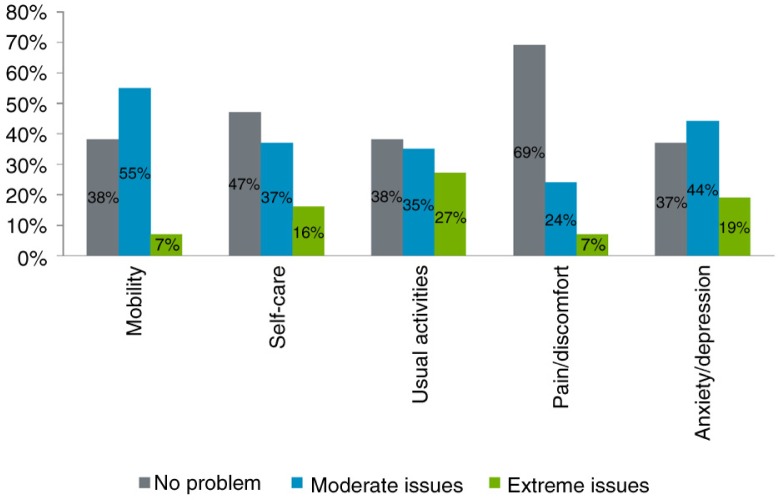
EQ-5D-3L dimensions scores.

Patients were also asked to complete the generic EQ-5D-3L questionnaire. Based on patients’ reports, usual activities and anxiety/depression were the most severely altered dimensions with 27% of patients reporting having extreme issues in performing usual activities and 63% of them having moderate to extreme issues of anxiety or depression. Mobility was also altered to a great extent: 62% of patients had moderate to severe motor problems. On the contrary, pain was the least altered dimension with 69% of patients having no problem of pain or discomfort (Fig. 4). The mean EQ-5D-3L utility was 0.54 (SD=0.43). EQ-5D-3L utilities decreased with disease severity, from 0.25 for a score of 60 score to 0.84 for scores above 80.

### Correlations between H-QoL-I and EQ-5D-3L

Pearson's correlations between H-QoL-I and EQ-5D-3L scores are shown in Table 3. All correlations were moderate to high and statistically significant (*p<*0.01), with the exception of the correlation between H-QoL-I socialising score and EQ-5D-3L anxiety score (*p=*0.1174) (Table 3). The highest correlations were found between H-QoL-I motor score and three EQ-5D-3L scores: mobility, self-care, and usual activity. Also, the H-QoL-I socialising score was highly correlated with the EQ-5D-3L usual activity score.

**Table 3:** Pearson's correlations

	EQ-5D-3L utility scores				
	
Item	Mobility	Self-care	Usual activities	Pain/discomfort	Anxiety/depression
H-QoL-I motor functioning score	**0.69**	**0.78**	**0.78**	0.48	0.38
H-QoL-I psychology score	0.44	0.38	0.49	0.41	0.44
H-QoL-I socialising score	0.58	0.56	**0.63**	0.46	0.22
H-QoL-I total score	**0.67**	**0.67**	**0.73**	0.52	0.43

Figures in bold are correlations>0.65.

## Discussion

HD is a devastating illness because of its steady ravaging progression and impact on families, leading to sustained disability for patients and poor quality of life. Understanding HRQoL in HD is necessary to evaluate the effectiveness of clinical interventions.

In this first study to assess the HRQoL of Spanish patients with HD, we used the generic EQ-5D-3L and the specific H-QoL-I instruments. With regards to previous studies, both instruments confirmed that HD patients have significant limitations in their HRQoL, particularly in the motor and psychological dimensions. The majority of patients not only reported having difficulties in performing motor tasks, such as tying their laces, but also felt anxious or depressed because of the disease. On the contrary, pain and social dimensions were shown to be less affected by the disease (Figs. 2 and 4).

The specific H-QoL-I instrument, especially developed for this study, spans the three crucial domains that assess the HRQoL of patients with HD. Although both instruments were able to assess patients’ HRQoL, there were some discrepancies in the extent of affected dimensions between the two instruments. Although usual activities, mobility, and anxiety/depression were the most severely altered dimensions according to the EQ-5D-3L instrument (Fig. 3), H-QoL-I was more able to capture the psychological dimension of the disease with more specific items targeting this dimension. Patients reported the feeling of powerlessness and the worry about symptoms of their disease (Fig. 2). Pearson's correlations also demonstrated that the two instruments measure slightly different HRQoL attributes: H-QoL-I psychology score and H-QoL-I socialising scores did not correlate to any dimension of the EQ-5D-3L instrument (Table 3). The highest correlations were found between H-QoL-I motor functioning score and two EQ-5D-3L scores: self-care and usual activity.

The specificity of the H-QoL-I begins in the items of the motor functioning dimension, which are the most relevant to the handicap caused by HD as well as in the items relating to the patient's acceptance that there will be no possible improvement in their health status (feeling of powerlessness). The main advantage of the H-QoL-I versus a generic HRQoL instrument is, therefore, that it takes into account the particular aspects of the disease.

Generic measures such as EQ-5D-3L have the advantage of allowing comparisons to be made between different illnesses and the impact they have on the quality of life of patients. However, generic HRQoL instruments do not fully capture the triad of symptoms typical of HD. Helder et al. highlighted the fact that there was a lack of differentiation between illness-related variables associated with physical and mental (i.e., psychosocial) HRQoL using the SF-36 instrument (8). The authors insisted on the importance of working towards creating a disease-specific instrument for HD, which would be better suited to capturing the unique constellation of HD signs and symptoms on HRQoL.

The H-QoL-I was specifically developed with the aim of addressing these shortcomings, and this study showed that this instrument was able to adequately capture HD-related variables that other generic instruments were not able to differentiate, especially the psychological dimension.

A number of shortcomings of this study should be mentioned. First, the sample size of Spanish participants was relatively small but could be considered acceptable given the prevalence of HD in Europe. Second, the study design could potentially lead to a biased selection because of recruitment of only patients followed by a neurologist. Also, the generalisation of the results could be questionable as only one centre was included; however, similar results were found in the other countries that participated in this study (20). Another limitation of the study was its lack of longitudinal data. Comprehensive, large-scale longitudinal studies will be necessary to further describe the burden and the HRQoL of HD patients and their caregivers.

In conclusion, this study demonstrated that the quality of life of the Spanish HD patients was seriously affected by the disease. Motor and psychological dimensions were the most affected, whereas pain and social dimensions were the least affected. Better therapeutic options to improve motor function and thereby HRQoL are needed.
